# TDCS effects on pointing task learning in young and old adults

**DOI:** 10.1038/s41598-021-82275-4

**Published:** 2021-02-09

**Authors:** E. Kaminski, M. Engelhardt, M. Hoff, C. Steele, A.Villringer, P. Ragert

**Affiliations:** 1 Institute for General Kinesiology and Exercise Science, Faculty of Sport Science, University of Leipzig, Jahnallee 59, 04109 Leipzig, Germany; 2 Department of Neurology, Max Planck Institute for Human Cognitive and Brain Sciences, Leipzig, Germany; 3 Einstein Center for Neurosciences, Charite-Universitätsmedizin Berlin, Berlin, Germany; 4 Department of Neurosurgery, Charite-Universitätsmedizin Berlin, Berlin, Germany; 5 Department of Psychology, Concordia University, Montreal, QC Canada; 6 Berlin School of Mind and Brain, Humboldt-Universität zu Berlin, Berlin, Germany; 7 Charité-Universitätsmedizin, Berlin, Germany

**Keywords:** Motor cortex, Consolidation, Short-term memory, Cognitive ageing

## Abstract

Skill increase in motor performance can be defined as explicitly measuring task success but also via more implicit measures of movement kinematics. Even though these measures are often related, there is evidence that they represent distinct concepts of learning. In the present study, the effect of multiple tDCS-sessions on both explicit and implicit measures of learning are investigated in a pointing task in 30 young adults (YA) between 27.07 ± 3.8 years and 30 old adults (OA) between 67.97 years ± 5.3 years. We hypothesized, that OA would show slower explicit skill learning indicated by higher movement times/lower accuracy and slower implicit learning indicated by higher spatial variability but profit more from anodal tDCS compared with YA. We found age-related differences in movement time but not in accuracy or spatial variability. TDCS did not facilitate learning neither in explicit nor implicit parameters. However, contrary to our hypotheses, we found tDCS-associated higher accuracy only in YA but not in spatial variability. Taken together, our data shows limited overlapping of tDCS effects in explicit and implicit skill parameters. Furthermore, it supports the assumption that tDCS is capable of producing a performance-enhancing brain state at least for explicit skill acquisition.

## Introduction

Motor skill learning is an essential part of our everyday life, ranging from experts performance in sports^1^ or music^2^ to recovery of motor functions after pathological brain lesions^3^. Skilled motor performance can be measured as task success, an often explicit categorical measure, but also via more implicit continuous measures of movement kinematics^4^. Task success in motor learning studies is mainly characterized by performance parameters such as speed, accuracy or efficiency^5–8^ measured by accuracy rates, reaction times or the trade-off between both parameters^7–10^, which are usually reported as a single value per trial. Measures of movement kinematics allow the characterization of a variety of parameters such as position, velocity, acceleration or movement direction^11^ but also temporal and spatial characteristics of motor output variability^12–16^. Variability in motor movement performance stems from a variety of different adaptational processes, including integration of sensory feedback^17^. In line with the approach that unstable sensory conditions need to be integrated into internal models^17,18^, motor variability can be considered as noise in the neuro-motor system causing variability in motor output performance^16,19^. Learning a new motor movement includes variability reduction in motor performance^10^ but also for example implicit learning of spatial accuracy^20^ or learning the best task-specific strategy^21^. Even though parameters of task success such as accuracy and movement kinematics are often related, there is evidence that they represent distinct concepts of learning with regard to its explicitness^4,22^. Explicit and implicit components of learning are likely to be maintained in separate but interacting systems^20,23,24^. Therefore, investigating whether learning a novel motor skill task is associated with both increases in explicit task success and more implicit decreases in motor output variability might help understanding the relationship between both explicit and implicit learning components.

Aging is one major factor modulating motor skill learning^25–30^. Presumably, one reason is that aging profoundly impacts the sensorimotor network^31–34^, resulting in a progressive decline in motor functions^32,35–38^. Learning a novel motor task in older age involves a much more distributed neural network compared with younger adults (YA)^39,40^. This phenomenon, however, cannot merely be attributed to increased neural noise but presumably serves as a necessary compensatory mechanism for successful task performance in older age^39–41^. Even though there is evidence showing intact explicit skill learning in older adults (OA)^25,27^, rates of motor learning are usually smaller^26,28^. Therefore, extended practice periods and different strategies are necessary to obtain similar explicit skill levels as younger cohorts^27,29,42^. Furthermore, there is some evidence suggesting that OA perform motor tasks with higher motor variability^38,43,44^. Age-related increase in variability may be caused by age-related changes in motor unit morphology and properties^38^ but may furthermore also represent a decreased ability to flexibly adapt to varying task constraints^43^. In this regard, adding kinematic measures in motor learning investigations in OA may help unraveling more implicit mechanisms of motor control in the learning process.

In recent years, non-invasive brain stimulation techniques such as transcranial direct current stimulation (tDCS) became popular with the potential to improve motor performance and learning in a variety of different motor tasks (for review see^45^). Mechanistically, tDCS is capable of enhancing motor excitability^46^ at postsynaptic cortical neurons^47–49^. Since long-lasting synaptic potentiation^50^ is considered a mechanism of plasticity, tDCS combined with motor practice seems to be capable of systematically augmenting brain function. The primary motor cortex (M1) is one of the key regions involved in the motor learning process^7^ showing reduced activity with extended practice^51^, which is why anodal tDCS over M1 has been predominantly used to facilitate early skill acquisition^52,53^. The facilitatory effect of M1 tDCS was shown in sequential motor tasks^52,54^ and continuous cursor navigation tasks, for example during continuous force transduction^55^, joint flexion and extention^56,57^ or reaching movements^58^ but mainly for explicit learning parameters such as accuracy or movement time. Interestingly, it seems that enhancing cortical excitability via M1 tDCS does not influence the motor act per se but only those synaptic connections, previously selected by training, indicating specificity in learning with regards to the sequence or the movement^59^. However, since there is some evidence suggesting that tDCS effects also transfer to unlearned aspects of motor tasks^60^, one can assume that the amount of transfer depends on the underlying function of the stimulated brain structure and therefore on the motor task that is performed. Furthermore, M1 plays a major role in consolidating a learned motor movement. In fact, skill acquisition over multiple days is mainly enhanced via an effect of tDCS on consolidation^9,54,61^, indicated by major increases in offline gain scores. Interestingly, over multiple tDCS sessions, offline gain enhancement accumulates, leading to observable effects on long-term retention up to months after the experimental session^9,54,61^. In OA, tDCS over M1 has also been used to improve motor learning rates^62–64^ and as one study indicates, tDCS may even produce greater improvement than in YA^65^. Authors mainly attribute this effect to a larger room for improvement, which is also supported by a positive association between age and skill improvement^64^. Since M1 stimulation is also capable of facilitating motor memory consolidation in OA^66^, one may speculate that multiple tDCS sessions in OA have the potential to produce similar effects to those seen in YA^9^.

In the present study, we aimed to investigate the effects of multiple tDCS-sessions on an arc pointing task (APT) in YA and OA. APT can be considered a complex motor skill^4^ that requires highly precise pointing movements which allow the analysis of specific kinematics on a single movement basis. Furthermore, since APT learning is associated with increased activation in M1^67^, it provides a suitable target for modification with tDCS. Anodal tDCS was applied over M1 during APT training on 3 consecutive days to investigate tDCS-induced improvements in both explicit and implicit measures of motor performance^53^. We hypothesized that (i) OA would perform APT with lower accuracy and higher movement times and also show smaller learning rates^25,68^. Furthermore, we also expect them to show higher spatial variability compared to YA, indicating also lower implicit learning. Since lower performance values at baseline provides greater room for improvement, we hypothesized that (ii) OA would show greater gain than YA in both explicit and implicit measures of learning as a result of tDCS. Furthermore, we hypothesized that (iii) multiple tDCS-sessions will mainly enhance offline gain^9,54,61^ in a movement-specific way, suggesting that only learning in trained but not transfer movements are facilitated.

## Results

### Demographics

Age groups did not differ regarding demographic variables besides age (see Table 1 for details). All participants tolerated the stimulation well and none reported any unexpected side effects from tDCS stimulation. Across both age groups, a chi-squared test revealed no difference between members of the a-tDCS and s-tDCS group in the ability to judge their group belonging (χ^2^(1) = 0.29, p = 0.59), indicating that the blinding of conditions was effective. Additionally, differences between pre and post experimental ratings of attention, fatigue and discomfort did not differ between age groups nor between stimulation groups.

**Table 1:** Sample characteristics.

Group	Age (years)	Gender (f/m)	LQ	Regular video gaming	Joystick experience
YA (n = 30)	27.7 ± 3.8	17/13	83.3 ± 13.98	11/30	9/30
OA (n = 30)	67.97 ± 5.32	15/15	82.7 ± 25.3	9/30	8/30

Age groups did not differ regarding their laterality quotient (LQ), independent-samples t-test: t(45.21)  = 0.12, p = 0.91), the amount of regular video gamers (Chi-square test: χ^2^(1) = 0.07, p = 0.79) and the amount of people with joystick experience (Chi-square test: χ^2^(1) = 0.08, p = 0.77).

### Aging effects on APT learning

**Figure 1:**
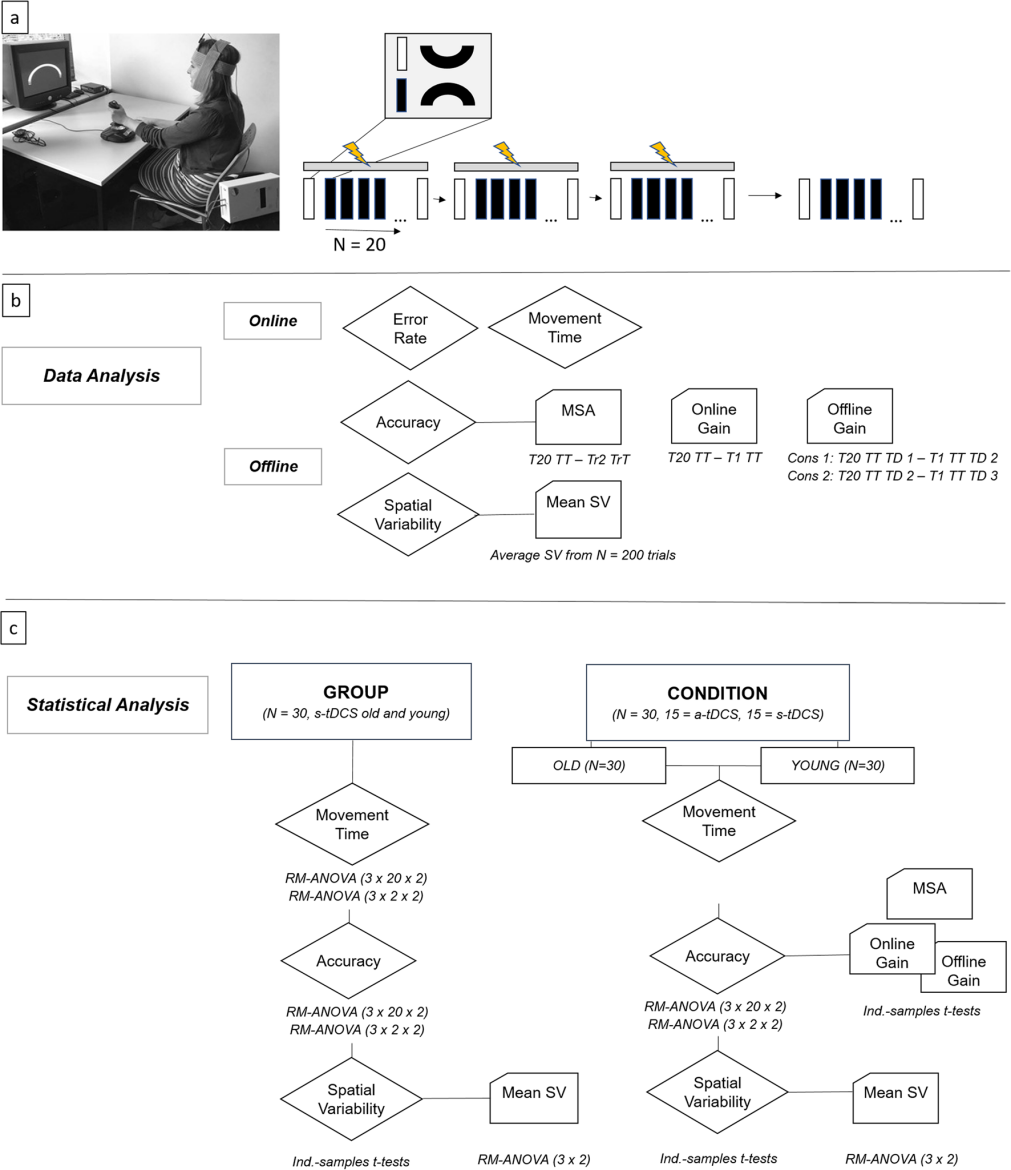
(**a**) Study design. Participants sat facing a computer screen with their left wrist on a joystick while they received 20 min of a-tDCS or s-tDCS over the contralateral primary motor cortex. Their main task was to move the cursor from the start to the target box through the arc channel without exceeding the channels boundaries as fast and as accurately as possible. The study consisted of three consecutive training days with tDCS (indicated by yellow flash) seperated by 24 h and a retention day without tDCS 1 week later. Each training day consisted of 20 learning (black rectangle) and 2 transfer trials (white rectangle). Learning trials included upward movements in clockwise direction, transfer trials downward movements in counter-clockwise direction. (**b**) Data analysis. MSA: movement specific accuracy, TT: training trial, TrT: transfer trial, TD1: training day 1, TD2: training day 2, TD3: training day 3, SV: Spatial Variability. Online calculations included movement time and error rate calculation. Offline calculations included accuracy calculation (1-error rate) and calculation of SV (variability of time-normalized radial position data). Furthermore, subparameters of accuracy were calculated: MSA corresponds to the absolute difference between last TT (T20) and second TrT (Tr2). Online gain scores were calculated as difference between T20 TT and T1 TT. Offline gain scores were calculated as differences between last and first TT of consecutive TDs, values > 0 represent skill consolidation and values < 0 represent skill loss. (**c**) Statistical analysis. RM-ANOVA: repeated-measures analysis of variances, ind-samples t-tests: independent samples t-tests.

Movement time (3 × 20 × 2 Repeated Measures-Analysis of variance (RM-ANOVA)) decrease in training trials (TT) over time was not different across age groups (interaction (IA) trial*day*group, F (5.93, 160.1) = 1.38, p = 0.23, η_p_^2^ = 0.05). Movement time also decreased within each training day (main effect (ME) trial, F (4, 160.1) = 1.35, p = 0.26, η_p_^2^ = 0.05) but interestingly not across training days (ME day, F (1.64, 160.1 = 0.003, p = 0.99, η_p_^2^ = 0). Generally, OA exhibited higher movement time values than YA (ME group, F (1,27) = 38.7, p < 0.001, η_p_^2^ = 0.59), see also Fig. 1. In transfer trials (TrT), movement time (3 × 2 × 2 RM-ANOVA) did not decrease within training days (TDs) (ME trial, F (1, 54) = 1.26, p = 0.27, η_p_^2^ = 0.05) but between training days (ME day, F (1.48, 49.7) = 3.99, p = 0.04, η_p_^2^ = 0.13). Furthermore, movement time was higher in OA than in YA also in TrT (ME group, F (1,27) = 13.46, p = 0.001, η_p_^2^ = 0.33) but the decrease over time was not modulated by age (IA trial*day*group, F (2,54) = 0.25, p = 0.78, η_p_^2^ = 0.01).

Accuracy (3 × 20 × 2 RM-ANOVA) increase in TT over time was not modulated by age (IA trial*day*group, F (13.77, 358.18) = 0.65, p = 0.95, η_p_^2^ = 0.02), however across both age groups accuracy significantly increased within (ME trial, F (8.03, 358.18) = 7.97, p < 0.001, η_p_^2^ = 0.24) and also across all training days (ME day, F (1.36, 358.18) = 13.28, p < 0.001, η_p_^2^ = 0.34). Furthermore, accuracy in TT did not differ across age groups (ME group, F (1,26) = 0.81, p = 0.38, η_p_^2^ = 0.03), see also Fig. 1. Also in TrT, accuracy (3 × 2 × 2 RM-ANOVA) changes over time were not modulated by age (IA trial*day*group, F (2,54) = 1.18, p = 0.32, η_p_^2^ = 0.04). TrT accuracy increased within (ME trial, F (1,49.65) = 6.2, p = 0.02, η_p_^2^ = 0.19) and across training days (ME day, F (1.44, 49.65) = 4.27, p = 0.03, η_p_^2^ = 0.14) and did not differ between age groups (ME group, F (1, 27) = 0, p = 1, η_p_^2^ = 0).

**Figure 2:**
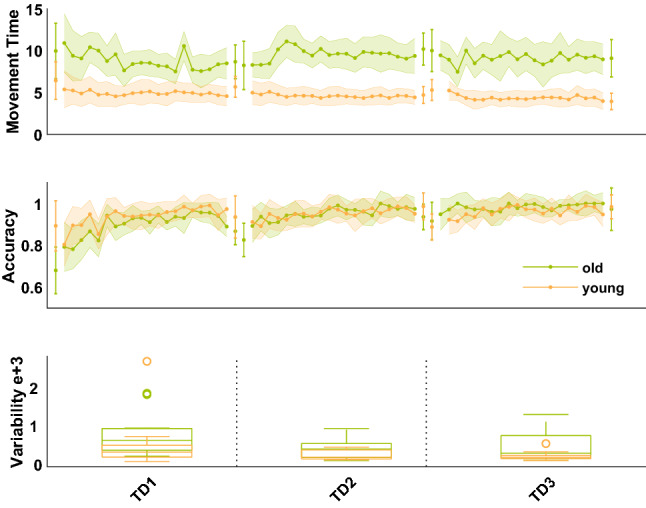
Line Graph shows median values ± 95% Confidence Interval. Green line represents OA, orange line represents YA.

Spatial variability did not differ between age groups in none of the time-normalized data points (all p-values > 0.05/200, bonferoni threshold: 0.00025). Mean spatial variability (3 × 2 RM-ANOVA) decreased over time (ME day, F (1.54, 43.25) = 3.65, p = 0.04, η_p_^2^ = 0.12), but the variability decrease was not modulated by group (IA day*group, F (1.54, 43.25) = 0.69, p = 0.47, η_p_^2^ = 0.02). We found no effect of age group on mean spatial variability (ME group, F (1,28) = 3.03, p = 0.09, η_p_^2^ = 0.1), even though descriptively, OA showed higher higher spatial variability (525.43 ± 99) than YA (336.45 ± 198), see also Fig. 2.

### TDCS effects on APT learning

#### OA

Movement time (3 × 20 × 2 RM-ANOVA) decrease in TT was not affected by tDCS (IA trial*day*condition, (F (8.52, 213.08) = 1.13, p = 0.35, η_p_^2^ =  0.04, BF_10_ = 0.15). Generally, average movement times did not differ across stimulation conditions (ME condition, F (1,25) = 0.1, p = 0.76, η_p_^2^ = 0.004, BF_10_ = 0.48) indicating no effect of tDCS on movement times. Also in TrT (3 × 2 × 2 RM-ANOVA), tDCS did not affect movement times (ME condition, F (1, 26) = 0.01, p = 0.91, η_p_^2^ = 0 .001, BF_10_ = 0.43) and also movement time decrease over time did not differ across stimulation conditions (IA trial*day*condition, (F (2, 52) = 0.04, p = 0.96, η_p_^2^ =  0.002, BF_10_ = 0.16).

**Figure 3:**
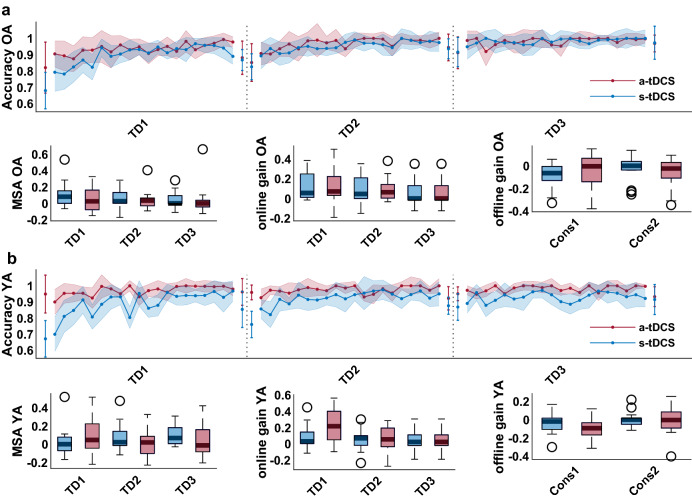
(**a**) OA. (**b**) YA. Line Graph shows median values ± 95% Confidence Interval. Red line represents a-tDCS group, blue line represents s-tDCS group. Separated values represent transfer trials, connected lines represent training trials. Dotted lines indicate separation of training days. Boxplots show median with 25th and 75th percentiles. Outliers are depicted as dots. Red boxes represent the a-tDCS group, blue boxes represent the s-tDCS group.

Accuracy in TT (3 × 20 × 2 RM-ANOVA) was not affected by tDCS (IA trial*day*condition, (F (38, 950) = 1.25, p = 0.14, η_p_^2^ = 0.05, BF_10_ = 0.008, ME condition, F (1,25) = 0.69, p = 0.42, η_p_^2^ = 0.03, BF_10_ = 0.377), see also Fig. 3a. Also TrT accuracy (3 × 2 × 2 RM-ANOVA) was not modulated by tDCS (IA trial*day*condition, F (1,26) = 0.06, p = 0.81, η_p_^2^ = 0.002, BF_10_ = 0.24, ME condition, F (1,26) = 0.06, p = 0.81, η_p_^2^ = 0.002, BF_10_ = 0.39). Movement specific accuracy (MSA, see Fig. 2 for details on calculation) was not affected by tDCS (MWU, TD1: U = 138, p = 0.3, BF_10_ = 0.56; TD2: U = 121, p = 0.74, BF_10_ = 0.35; TD3: U = 120, p = 0.52, BF_10_ = 0.41) and also online gain scores did not differ between stimulation conditions (TD 1: MWU, U = 121.5, p = 0.47, BF_10_ = 0.42, TD 2: MWU, U = 121, p = 0.72, BF_10_ = 0.37 TD 3: MWU, U = 121, p = 0.72, BF_10_ = 0.36). Furthermore, offline gain scores did not differ neither between TD 1 and 2 (consolidation 1: MWU, U = 138.5, p = 0.28, BF_10_ = 0.53) nor between TD 2 and 3 (consolidation 2: MWU, U = 99, p = 0.58, BF_10_ = 0.42), see also Fig. 3a.

**Figure 4:**
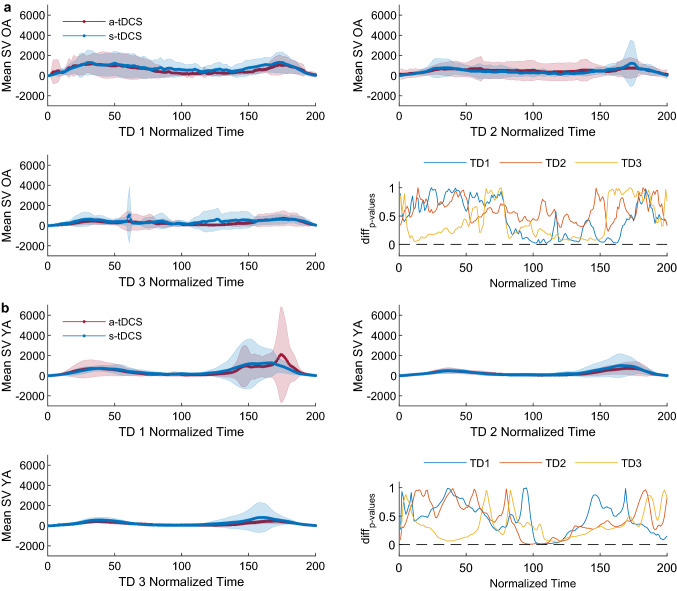
(**a**) OA. (**b**) YA. Plots 1–3 show spatial variability across all training days, lines represent mean values ± standard deviation. Plot 4 shows p-values for each of the 200 t-tests across all three training days investigating group differences in spatial variability of the cursors’ radial position (solid lines: blue: day 1, orange: day 2, yellow: day 3) as well as the p-value threshold corrected for multiple comparisons (dashed line, bonferoni-corrected p-value = 0.00025).

Spatial variability did not differ between stimulation conditions in none of the time-normalized data points (all p-values > 0.05/200, bonferoni threshold: 0.00025), see Fig. 4a. Also regarding mean spatial variability (3 × 2 RM-ANOVA), we found no effect of the stimulation condition (ME condition, F (1, 28) = 1.09, p = 0.31, η_p_^2^ = 0.038, BF_10_ = 0.47) and also the amount of variability reduction did not differ across conditions (IA day*condition, F (1.63, 45.78) = 1.27, p = 0.29, η_p_^2^ = 0.04, BF_10_ = 0.41).

#### YA

Movement time (3 × 20 × 2 RM-ANOVA) decrease in TT was not affected by tDCS (IA trial*day*condition, (F (38, 1064) = 1.35, p = 0.08, η_p_^2 =^ 0.05, BF_10_ = 0.004). Generally, average movement times did not differ across stimulation conditions (ME condition, F (1, 28) = 4.05, p = 0.05, η_p_^2 =^ 0.13, BF_10_ = 1.55). Also in TrT (3 × 2 × 2 RM-ANOVA), tDCS did not affect movement times (ME condition, F (1, 28) = 0.65, p = 0.43, η_p_^2 =^ 0.02, BF_10_ = 0.53) and also movement time decrease over time did not differ across stimulation conditions (IA trial*day*condition, (F (1.36, 38.18) = 0.32, p = 0.64, η_p_^2 =^ 0.01, BF_10_ = 0.22).

TDCS did not affect accuracy (3 × 20 × 2 RM-ANOVA) learning in TT (IA trial*day*condition, F (38, 1064) = 1.006, p = 0.46, η_p_^2^ = 0.04, BF_10_ = 0.004). However, accuracy was higher in the a-tDCS compared with s-tDCS group in TT (ME condition, F (1, 28) = 15.65, p < 0.001, η_p_^2^ = 0.36, BF_10_ = 39.45), already starting from the first learning trial (t (25.57) = 2.68, p = 0.013, BF_10_ = 4.39), see also Fig. 3b. To evaluate potential baseline differences between conditions in more detail, the RM-ANOVA was rerun using the accuracy value from the first transfer trial (TrT 1) as a covariate. Here, we still found higher accuracy values in a-tDCS compared with s-tDCS conditions (ME condition, F (1, 27) = 10.91, p = 0.003, η_p_^2^ = 0.29, BF_10_ = 18.47) but still no difference in accuracy increase over time (IA trial*day*condition, F (38, 1026) = 0.94, p = 0.58, η_p_^2^ = 0.03, BF_10_ = 0.004). Accuracy differences between conditions were still present at the retention session 1 week after the last training session (ME condition, F (1,28) = 5.19, p = 0.03, η_p_^2^ = 0.16, BF_10_ = 1.62). Interestingly, TrT accuracy (3 × 2 × 2 RM-ANOVA) did not differ between conditions (ME condition, F (1, 28) = 0.9, p = 0.35, η_p_^2^ = 0.03, BF_10_ = 0.44), also not over time (IA trial*day*condition, F (2, 56) = 0.64, p = 0.53, η_p_^2^ = 0.02, BF_10_ = 0.26). MSA was not affected by tDCS (MWU, TD1: U = 85, p = 0.26, BF_10_ = 0.45; TD2: U = 131.5, p = 0.44, BF_10_ = 0.47; U = 155, p = 0.08, BF_10_ = 1.03) and also regarding online gain, no tDCS effects were found (TD 1: MWU, U = 152, p = 0.1, BF_10_ = 0.82, TD 2: MWU, U = 123, p = 0.66, BF_10_ = 0.35, TD 3: MWU, U = 116, p = 0.86, BF_10_ = 0.35). Furthermore, consolidation was not affected by tDCS (consolidation 1: MWU, U = 70, p = 0.08, BF_10_ = 1.05, consolidation 2: MWU, U = 103, p = 0.7, BF_10_ = 0.37).

Spatial variability of each time-normalized data point did not differ between stimulation groups (all p-values > 0.05/200, bonferoni threshold: 0.00025, see also Fig. 4b), indicating no group difference in spatial variability. Mean spatial Variability (3 × 2 RM-ANOVA) was comparable across tDCS conditions (ME condition, F (1, 28) = 0.47, p = 0.5, η_p_^2^ = 0.02, BF_10_ = 0.37) and we found no difference in variability reduction depending on the stimulation condition (IA day*condition, F (1.12, 31.39) = 0.13, p = 0.75, η_p_^2^ = 0.01, BF_10_ = 0.38), see also Fig. 4b.

## Discussion

The present study investigated the effect of multiple tDCS sessions over the M1 hand area on explicit and implicit measures of motor skill learning comparing YA and OA. Our results revealed that both age groups were able to learn the APT. While movement time reduced and accuracy increased over time, spatial variability decreased over time, indicating both explicit and implicit APT learning. As hypothesized, OA showed higher movement times than YA, while the amount of reduction over time did not differ, indicating similar learning rates. Contrary to our hypothesis (i), accuracy and spatial variability did not differ between YA and OA. Furthermore, against our hypothesis (ii), tDCS did not affect offline nor online APT learning in either group. However, in YA, accuracy was higher in the a-tDCS group compared with s-tDCS. This enhancement in accuracy, however, was not restricted to the learned movement since MSA was not affected by the stimulation and was also not accompanied by a related reduction in spatial variability. We also did not find a cumulative effect of multiple stimulation sessions, as hypothesized in (iii), which has previously been described in similar multi-session tDCS learning studies^9,54^.

Our results are in line with previous findings showing that APT-learning is associated with improvements in accuracy^4^. However, participants showed much higher accuracy than those of the original study, and longer movement times. We found mean movement times between three to six seconds, while in the previous study participants trained at a medium movement time range of 620 ms^4^. Since Shmuelof et al.^4^ showed a generalization of skill learning across all included movement time categories, we assumed that it is not necessary to restrict movement times to a certain range but instruct participants to perform as fast as possible. However, we suspect participants were not aware of how fast they could perform the task and therefore performed at a convenient speed level. Participants’ strategy may have been to prioritize error avoidance over speed, which is potentially why high accuracy values were observed. This behavior may have altered the relative duration of learning stages^7^ but also the relative recruitment of brain regions^69–71^. Furthermore, in young participants, accuracy values were close to 100 percent at the end of the training sessions. The missing effect of tDCS-induced APT skill learning could be attributed to the fact, that participants performed at their peak performance (ceiling) level. However, since we observed a main effect of stimulation and no initial ceiling was observed, we argue, that further improvement was still possible.

Furthermore, our findings underline the importance of investigating motor skill acquisition in different cohorts since learning strategies may differ tremendously between age groups. It is well known that aging is associated with a progressive decline in motor functioning with evidence for reaction or response time slowing^27,36,72^, diminished accuracy in movement execution^30^ and increased motor output variability^38,43,44,73^. Different age groups have been shown to use different strategies to learn novel tasks especially during the fast initial phase of learning^74,75^ and compensatory strategies in aged individuals such as slowing of movement time are especially relevant for perceptual and higher-order cognitive processing^29,42,76^. Our data supports these findings showing that OA perform the APT with slower reaction times and potentially thereby increase accuracy up to a level of YA^29^. Thus, one could speculate that high levels of accuracy in OA are achieved with a different behavioral strategy than YA. In line with this argument are the different trajectory variability courses visible in YA and OA, even though mean spatial variability was not affected by age. While OA show similar spatial variability throughout the movement, YA show greater variability at the end of the movement. Shmuelof and colleagues interpret decreases in trajectory variability around the average path as improvements in feedforward control, while trajectories with large deviations at the end of the movement represent feedback improvements^4^. Following this idea, OA APT performance may improve mostly from information *during* the motor task (knowledge of performance) for example via immediate feedback of the cursor position. YA, instead, may rather profit from information *after* task performance for example via feedback on movement time and error rates (knowledge of result). However, this needs to be explicitly tested in future studies examining the effect of different feedback conditions on APT learning in different age cohorts. Age-related alterations on motor behavior are related to functional and structural alterations on a brain level including grey and white matter loss but also differential neural activation patterns^30,77–83^. It cannot be ruled out that APT learning in OA is not primarily associated with M1 activation but rather relies on higher-order brain regions such as (pre)-frontal cortices. This question needs to be addressed by future studies, which should aim at identifying brain regions associated with APT learning in OA to unravel potential targets for the application of non-invasive brain stimulation. Furthermore, aging also changes the propensity for plasticity modulation^84,85^, which may limit the potential of tDCS to induce a facilitatory learning effect in OA. However, tDCS over M1 did not facilitate APT learning in YA either. Therefore, methodological issues such as duration or time point of the stimulation but also insufficient current intensity could be held responsible for the limited tDCS effect on learning^86^. Our current density (0.028 mA/cm^2^) was a little bit lower as the one previously used^9,54,61^ which may have reduced tDCS efficacy^87^. In contrast, stimulation duration was set to 20 min which falls within the range, commonly used^9,54^. Furthermore, also subject factors such as biological variation but also the current neuronal state of the target region influence the responsiveness to tDCS^88^, making direkt monocausal inferences from missing tDCS effects on learning impossible. Similar multi-session tDCS learning studies^9,54^ mainly found a cumulative M1 tDCS effect on offline learning gains, highlighting the importance of M1 in early consolidation. In our study design, no tDCS effect on offline learning was found, contradicting previous findings. Additional analyses using BF indicate inconclusive evidence to interpret this finding as a null effect. One potential explanation for the divergent results between previous and our study may originate from differences in the motor task since the exact role of M1 differs depending on the type of motor learning task being learned^45,54^. APT learning is mainly associated with the reduction of variability of a motor action. In this regard, acquisition of task-specific synergies, which mainly update *during* task performance, could have mainly happened in M1^67^, explaining at least the missing tDCS effect on offline learning performance. Furthermore, a previous study interpreted the APT learning-related increase in M1 activation as a recruitment of additional neurons^67^. However, tDCS effects are mainly based on mechanisms enhancing synaptic plasticity^50^, thus may not be suitable for enhancing APT learning-associated recruitment processes within M1. Furthermore, the calculation of BFs in addition to conventional statistical analyses allow judgements for or against H0. However, even though BFs provide important information^89^, one has to keep in mind their negative aspects such that BFs are very sensitive to prior distributions, which can be too difficult to choose or depend on the belief that one true model exists, which may not always be the case^90^. Therefore, our assumption that our data provides only inconclusive evidence should be handled with caution. However, future studies in this field should nevertheless aim to use bigger samples to gain clear evidence for or against tDCS effects on offline APT accuracy learning.

We did see a difference in accuracy between both tDCS groups in YA, potentially indicating an involvement of M1 in APT performance at least in young age. BFs support this suggestion for a main stimulation effect on accuracy. However, even though we used appropriate randomization to select group membership, we cannot completely rule out that group differences already existed prior to tDCS since no baseline measurement was included to maintain task naivety. In favour of an existing effect, we hypothesize that M1-stimulation induced a performance-enhancing state which facilitated APT performance from the very beginning. This finding is consistent with Shmuelof et al., showing M1-activation from the beginning of APT learning^67^ and highlights the important role of M1 in initial skill acquisition in YA^7,91^. Brain activity prior to learning is undoubtedly an important predictor of subsequent motor performance^92^ and previous studies also found tDCS applied before learning facilitated subsequent skill acquisition rates^93–95^. By contrast, no tDCS-induced effect was found on spatial variability in either group. This finding contradicts our primary hypothesis that tDCS affects both explicit and implicit APT learning. We do see within-session and between-session reductions of spatial variability, indicating APT learning. In a taxonomy proposed by Krakauer and Mazzoni, M1 is mainly involved in variability reduction during skill learning^10^—which is in line with other research^67^. However, we found no evidence for M1 tDCS-induced reductions in spatial variability. One reason for this mismatch may be the the low statistical power induced by the relatively low number of participants but also the multiple testing. Looking at the p-value figure, it is observable that group-differences in spatial variability are closest to significance at position points 95–110, which indicate the turning point from an upwards to a downwards movement in the arc channel during the training trials. Thus, it could be speculated that lower spatial variability values at these critical turning points may have resulted in higher accuracy values in the a-tDCS group. However, since our data does not support this hypothesis, so far our results do not show that enhancing explicit parameters of task success such as accuracy necessarily correlate with differences in implicit parameters of movement execution such as spatial variability.

In summary, we provide novel evidence that APT learning occurs both in YA and OA but also reveal age-related differences in learning strategies related to higher movement times in OA. While no tDCS-induced differences in APT learning were observable over time, we did see a group effect of M1 tDCS on accuracy in YA from the very beginning, potentially indicating a tDCS-induced performance-enhancing brain state. However, tDCS-induced differences in accuracy values did not translate into tDCS-induced differences in spatial variability, indicating no necessary interaction between explicit and implicit APT learning. Mechanisms of action of tDCS-suppported motor training should be oberseved more carefully, while also considering age-related differences in motor learning abilities.

## Methods

### Sample characteristics

In total, 60 healthy individuals aged between 21 and 78 years (32 females) were enrolled in this double‐blinded, sham‐controlled study. 30 individuals were aged between 18 and 35 years (YA, mean age: 27.07 ± 3.8 years, 17 females) and 30 individuals were older than 55 years (OA, mean age: 67.97 years ± 5.3 years, 15 females). Participants were right-handed, indicated by a score > 40 in the Edinburgh Handedness Inventory^96^ and a similar number of people within each age group reported regular computer gaming and joystick eperience (see also Table 1). Highly-skilled participants such as professional musicians and athletes were excluded from participation. All participants gave written informed consent and the study procedures were approved by the local ethics committee of the University of Leipzig and conducted in accordance with the declaration of Helsinki. To exclude the presence of any neurological disease and/or contraindications relevant for the study procedures outlined below, all participants underwent a detailed neurological examination prior to the testing phase. Additionally, all participants were free of any medication affecting the central nervous system and were task naïve.

### Study design

The study comprised of four training days with the first three conducted on consecutive days separated by a 24-h break. Training day 4 was conducted 1 week after training day 3 to investigate potential effects on long-term retention. Before training day 1, participants were randomly allocated by a second experimentor to either receive 20 min of daily anodal tDCS (a-tDCS) or sham tDCS (s-tDCS), where the stimulator was put on for 30 s only to ensure blinding^52^. TDCS was put on five minutes prior to the start of the motor task. During the APT, participants performed 20 tt and two trt to investigate short-term transfer effects of each TD. On TD 4, participants performed another 20 learning and 2 transfer trials but no tDCS was applied (see also Fig. 2 for details).

### APT

#### Motor task

The APT^4^ required participants to move a cursor on the computer screen by moving a joystick with their left wrist. Similar to the original task, we also chose the left wrist in our right-handed study cohort to maximize the dynamic learning range. In all four training sessions, participants sat facing a computer screen where a semicircular channel was presented using the *Presentation* software (Neurobehavioral Systems, Inc., version 14.7). They were instructed to guide a cursor through the channel from one end to the other without exceeding the channels boundaries as fast and as accurately as possible (see also Fig. 4 for details, please also note that informed consent was obtained to publish the participants’ image in an online open access publication). Before the training started, participants familiarized themselves with the joystick by moving the cursor freely within a white square field on the screen. At the beginning of each trial, the semicircular channel was presented and participants were instructed to start the trial only when ready. As a result, the experiment was not considered to be a speeded reaction time task protocol. For starting the trial, participants had to move the cursor into the start box positioned on the left side of the channel. Entering this start zone initiated time-keeping and was indicated to the participant by a change in the start box colour to yellow. Time-keeping continued until the cursor reached the stop box on the right side of the channel. During TT, participants performed an upwards movement in clockwise direction followed by a downwards movement to enter the stop box. During TrT, a mirrored version of the channel was presented, where a downwards movement in a counter-clockwise direction was followed by an upwards movement to enter the stop box (see also Fig. 1). Maximum trial duration was set to 30 s. After this time interval, the trial stopped and was marked as incomplete.

#### Information on performance

Cursor position was visible throughout the whole movement. Thereby, participants received feedback about their current position in the channel. Additionally, after each trial, the trajectory of the cursor was projected on the screen. To maximize information gain, portions of the participant’s movements inside the channel were colored in white and portions outside the channel in red. Furthermore, total time (in seconds) and error (in percent of the fraction of movements outside the channel), were calculated online and displayed on the screen after each trial. Thereby, participants received knowledge of performance in the form of trajectory projections as well as knowledge of result by the information about time and error rates.

### Transcranial direct current stimulation (tDCS)

For tDCS, a weak direct current of 1 mA generated from a battery driven stimulator (neuroConn, Ilmenau, Germany) was delivered for 20 min via saline-soaked sponge electrodes. Stimulation was switched on five minutes prior to the start of training since a previous study suggested that this time period is sufficient to induce enduring excitability increases within M1^97^. Either a-tDCS or s-tDCS was applied to the right M1 contralateral to the left wrist. Stimulation was performed double-blinded to ensure unbiased results. Specifically, a second experimenter was responsible for controlling the stimulation but was otherwise not in contact with participants or the primary experimenter. To evaluate, whether blinding of conditions was effective, participants were asked to judge their group belonging after the third day of experimental testing by choosing between the options: “real stimulation” or “no stimulation”. The anatomical landmark for the right M1 hand area was identified with neuronavigation (Brainsight Version 2; Rogue Research, Montreal, QC, Canada) using the MNI coordinates (x, y, and z) 40, 20, and 54^98,99^. After localization with the neuronavigation system, the skin was prepared using alcohol pads to ensure good contact of the stimulation electrodes applied to the head. The impedance of stimulation electrodes was always kept below 10 kΩ for each participant. The anode (7 × 5 cm) was positioned over the right M1, the cathode (10 × 10 cm) was placed over the frontal orbit. Flexible elastic straps were used to additionally fixate the electrodes on the head. Current was ramped up for 30 s in the beginning of tDCS eliciting a transient tingling sensation on the scalp that faded over seconds and also ramped down for 30 s as described previously^52^. During s-tDCS, the current was increased, maintained and decreased for 30 s each. Before and after tDCS, participants rated their level of attention (1 = not attentive, 10 = very attentive), fatigue (1 = very fatiqued, 10 = no fatigue) and discomfort (1 = no discomfort, 10 = strong discomfort) on a visual analogue scale (VAS) to ensure potential differences between groups did not originate from differences in these global parameters.

#### tDCS current flow simulation

**Figure 5:**
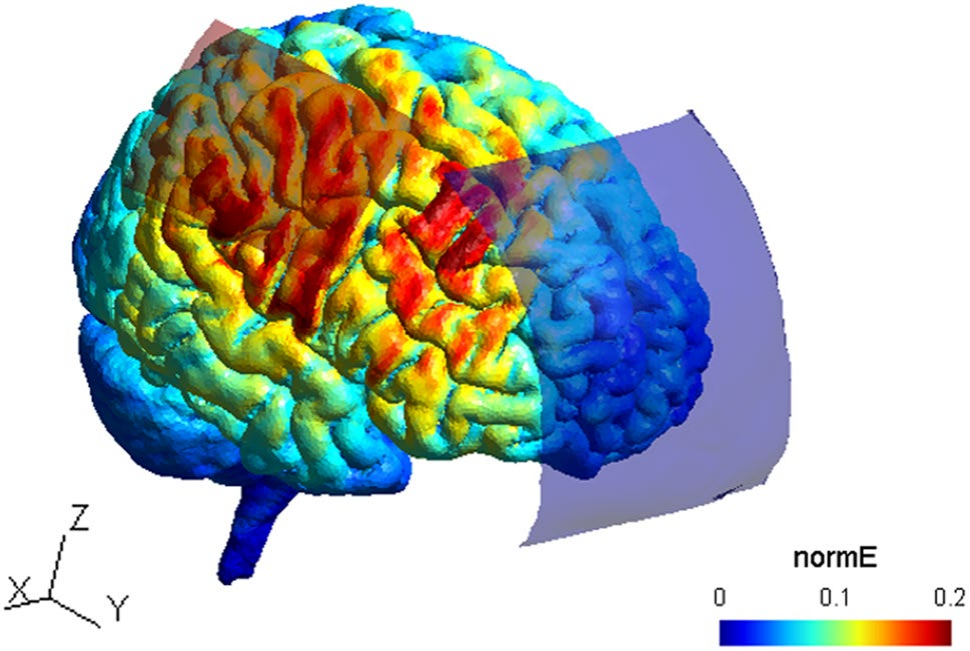
TDCS current flow simulation. Anode is depicted as red rectangle and cathode as blue rectangle projected on a standard head model. Normalized electrical field strength (V/m) is indicated through colormaps with blue representing lowest and red representing highest field strengths, respectively. The current flow image was created using the SIMNIBS software version 3.1.2^100^.

We simulated electric field distributions based on a finite element model of a representative head inside the open-source SimNIBS software^100^ to approximate current flow. The anode was defined according to our anatomical landmark (40, 20, 54 as x, y and z) with a size of 7 × 5 cm, the cathodes center position was defined at Fpz with a size of 10 × 10 cm. A current of 1 mA was selected. Maximum electrical field strength (0.2 V/m) was determined below the anode, corresponding to the hand area of right M1 but also in premotor areas between both electrodes (see Fig. 5).

### Data analysis

Data was analyzed both online and offline. Online analysis for movement time and error rates was performed by *Presentation* that controlled the experiment. Calculation of movement times was done by counting the number of data points captured from start to stop signal multiplied with 13.333 ms (sampling rate: 75 Hz, 1 data point every 13.333 ms) divided by 1000. For error rate calculation, each captured data point was screened for being inside or outside the channel. The number of data points outside the channel divided by the total number of data points was then multiplied with 100 to compute percentage error rates. For offline analysis, we used custom routines written in MATrix LABoratory R2018B (MATLAB, The MathWorks, Natick, MA). For offline calculations, movement times and error rates were extracted from the logfiles created by *Presentation*. Accuracy rates were calculated as 1-error rate which corresponds to the percentage of fraction of movements in channel. For investigating potential stimulation effects on accuracy in more detail, we additionally calculated MSA as well as online and offline gain scores. MSA was calculated for each training day as the difference of the accuracy value of the last learning trial per training day and the second transfer trial accuracy (t20–tr 2), see also Fig. 2. Online gain scores were calculated for each training day representing within-day learning as the difference between first and last learning trial accuracy (t20–t1), while offline gain scores were calculated between training days analog to^9^ to investigate between-day consolidation effects.

Cursor position data was low-pass filtered (zero-lag, third-order Butterworth filter, cutoff frequency 14 Hz) analog to Shmuelof et al.^4^ and filtering was applied at the trial level to remove any artifacts of returning the joystick to the home position. Averaging was performed only for data values outside the start box, data points within the start box were discarded from the analysis. Trial-by-trial variability was calculated to investigate the effect of tDCS-combined practice on trajectory on the cursors time-normalized radial position data. Data was resampled to 200 evenly spaced data points and then variance and average radial position was computed for each subject and time point. To additionally investigate mean spatial variability, average radial position was averaged across all 200 data points, see also Fig. 2.

### Statistical analysis

Statistical analyses were performed using the Statistical Software Package for Social Sciences (SPSS Version 27, IBM, Armonk, NY, USA). Difference scores of pre-post ratings for the VAS score ratings were compared across all subgroups using the between-subject factors group (old, young) and condition (a-tDCS, s-tDCS) in a repeated-measures analyses of variance (RM-ANOVA).

Aging effects on APT learning were calculated using only the data of the s-tDCS groups (N = 15 young and N = 15 old). Effects of age on movement time and accuracy were calculated for tt using RM-ANOVAs with between-subject factor group (young, old) and within-subject factors day (TD1–3) and trial (t1–t20). Likewise, effects on transfer movement time accuracy were calculated using RM-ANOVAs, see also Fig. 2. To investigate whether spatial variability of radial position differed between groups, we performed independent-samples t-tests or the non-parametric equivalent for every normalized time point, resulting in n = 200 tests for each training day. For investigating aging effects on mean spatial variability, we additionally performed a RM-ANOVA with factor group (old, young) and factor day (TD 1–3).

TDCS effects were calculated for each age group, separately to be able to parcellate aging effects from stimulation effects and decrease the number of factors in the analysis. TDCS effects on movement time and accuracy were calculated for training trials using RM-ANOVAs with between-subject factor condition (a-tDCS, s-tDCS) and within-subject factors day (TD 1–3) and trial (t1–t20) for each age cohort. Likewise, effects on trt movement time and accuracy were calculated using RM-ANOVAs. Furthermore, MSA, online and offline gain scores were compared across conditions using independent-samples t-tests or the non-parametric equivalent in case of non-normal distribution. TDCS effects on spatial variability were also investigated using independent-samples t-tests or the non-parametric equivalent for every normalized time point. Mean spatial variability was compared across conditions using an RM-ANOVA with factor condition (a-tDCS, s-tDCS) and factor day (TD 1–3), see also Fig. 2c (Supplementary Information).

Partial eta-squared (ηp2) for ANOVA’s are provided as measures of effect size and used to aid in the interpretation of inferential statistics. To control for multiple comparisions, p-values were adjusted according to the false-discovery-rate^101^. Conventional inferential statistics analyses are used to quantify our research hypotheses (H1) against the null hypothesis (H0). However, since conventional significance testing does not allow to state evidence for H0^102^, non-significant outcomes provide no information whether they represent real null findings or inconclusive evidence for example due to low sample sizes. To add this information, Bayes Factors (BF_10_) were calculated and provided in addition to the inferential data, quantifying how well H1 predicts the empirical data relative to H0^103,104^. Following recent recommendations, we considered BF_10_ > 1 as evidence for H1 over H0 with values > 3 suggesting noteworthy evidence, while BF_10_ < 1 indicated evidence for H0 over H1 with values < 0.33 suggesting noteworthy evidence^105^. Furthermore, BF_10_s between 0.33 and 3 are considered inconclusive evidence for either hypothesis^105^. BF_10_ were calculated using the statistical software package JASP (Jeffrey’s Amazing Statistics Program^106^).

## Supplementary Information

Supplementary Information.

## Data Availability

The data that support the findings of this study are available on request from the corresponding author, E.K. The data are not publicly available due to data protection policies practiced at our institute, e.g. their containing information that could compromise the privacy of research participants.
