# Author Correction: TDCS effects on pointing task learning in young and old adults

**DOI:** 10.1038/s41598-021-88987-x

**Published:** 2021-05-04

**Authors:** E. Kaminski, M. Engelhardt, M. Hoff, C. Steele, A. Villringer, P. Ragert

**Affiliations:** 1 Institute for General Kinesiology and Exercise Science, Faculty of Sport Science, University of Leipzig, Jahnallee 59, 04109 Leipzig, Germany; 2 Department of Neurology, Max Planck Institute for Human Cognitive and Brain Sciences, Leipzig, Germany; 3 Einstein Center for Neurosciences, Charite-Universitätsmedizin Berlin, Berlin, Germany; 4 Department of Neurosurgery, Charite-Universitätsmedizin Berlin, Berlin, Germany; 5 Department of Psychology, Concordia University, Montreal, QC Canada; 6 Berlin School of Mind and Brain, Humboldt-Universität zu Berlin, Berlin, Germany; 7 Charité-Universitätsmedizin, Berlin, Germany

Correction to: *Scientific Reports* 10.1038/s41598-021-82275-4, published online 09 February 2021

The original version of this Article contained an error in the Abstract.

“TDCS did not skill learning facilitate learning neither in explicit nor implicit parameters.”

now reads:

“TDCS did not facilitate learning neither in explicit nor implicit parameters.”

The original Article has been corrected.

