# Supplementary material for: TDCS effects on pointing task learning in young and old adults
Source: Sci Rep. 2021 Feb 9;11:3421. doi: 10.1038/s41598-021-82275-4 (PMC7873227; doi:10.1038/s41598-021-82275-4)
Supplement: Supplementary file 1 — Supplementary Information. [file 41598_2021_82275_MOESM1_ESM.docx]

**TDCS effects on pointing task learning in young and old adults**

E. Kaminski^1,2*^, M. Engelhardt^3,4^, M. Hoff^2^, C. Steele^2,5^, A.Villringer^2,6,7^, & P. Ragert^1,2^

^1^ Institute for General Kinesiology and Exercise Science, Faculty of Sport Science, University of Leipzig, Germany, ^2^ Department of Neurology, Max Planck Institute for Human Cognitive and Brain Sciences, Leipzig, Germany, ^3^Einstein Center for Neurosciences, Charite-Universitätsmedizin Berlin, Germany, ^4^Department of Neurosurgery, Charite-Universitätsmedizin Berlin, Germany ^5^Department of Psychology, Concordia University, Montreal, Quebec, Canada, ^6^Berlin School of Mind and Brain, Humboldt-Universität zu Berlin, Berlin, Germany, ^7^Charité-Universitätsmedizin, Berlin, Germany.

*Correspondence:

Elisabeth Kaminski, Dr., Institute for General Kinesiology and Exercise Science, Faculty of Sport Science, University of Leipzig, Jahnallee 59, 04109 Leipzig, Germany, Phone: +49-341-97-31686, Email: elisabeth.kaminski@uni-leipzig.de

STABLE 1: Statistical summary aging effects on arc pointing task learning:

| **Parameter** | **Factor 1: Group main effect** | | | **Factor 2: Day main effect** | | | **Factor 2: Trial main effect** | | | **Interaction: Group x Day (x Trial)** | | |
| --- | --- | --- | --- | --- | --- | --- | --- | --- | --- | --- | --- | --- |
|  | ***F*** | ***p*** | ***η_p_^2^*** | ***F*** | ***p*** | ***η_p_2*** | ***F*** | ***p*** | ***η_p_^2^*** | ***F*** | ***p*** | ***η_p_^2^*** |
| Movement Time  *Training Trials*  *Transfer Trials* | **38.7** | **<.001** | **.59** | .003 | .99 | 0 | 1.35 | .26 | .05 | 1.38 | .23 | .05 |
|  | **13.46** | **.001** | **.33** | **3.99** | **.04** | **.13** | 1.26 | .27 | .05 | .25 | .78 | .01 |
| Accuracy  *Training Trials*  *Transfer Trials* | .81 | .38 | .03 | **13.28** | **<.001** | **.34** | **7.97** | **<.001** | **.24** | .65 | .95 | .02 |
|  | 0 | 1 | 0 | **4.27** | **.03** | **.14** | **6.2** | **.02** | **.19** | 1.18 | .32 | .04 |
| Mean Spatial Variability | 3.03 | .09 | .1 | **3.65** | **.04** | **.12** |  |  |  | .69 | .47 | .02 |

Note: *η_p_^2^*: partial eta-squared, significant values marked in bold.

STABLE 2: Statistical summary tDCS effects on arc pointing task learning older adults.

| **Parameter** | **Factor 1: Condition main effect** | | | | **Interaction: Condition x Day (x Trial)** | | | |
| --- | --- | --- | --- | --- | --- | --- | --- | --- |
|  | ***F*** | ***p*** | ***η_p_^2^*** | ***BF_10_*** | ***F*** | ***p*** | ***η_p_^2^*** | ***BF_10_*** |
| Movement Time  *Training Trials*  *Transfer Trials* | .1 | .76 | .004 | .48 | 1.13 | .35 | .04 | .15 |
|  | .01 | .91 | .001 | .43 | .04 | .96 | .002 | .16 |
| Accuracy  *Training Trials*  *Transfer Trials* | .69 | .42 | .03 | .38 | 1.25 | .14 | .05 | .01 |
|  | .06 | .81 | .002 | .39 | .06 | .81 | .002 | .24 |
| Mean Spatial Variability | 1.09 | .31 | .038 | .47 | 1.27 | .29 | .04 | .41 |
|  |  |  |  |  |  |  |  |  |
|  |  |  |  |  |  |  |  |  |
|  |  |  |  |  |  |  |  |  |
|  |  |  |  |  |  |  |  |  |
|  |  |  |  |  |  |  |  |  |
|  | **Mean comparison:**  **(a-tDCS, s-tDCS)** | | |  |  |  |  |  |
|  | ***U*** | ***p*** | ***BF_10_*** |  |  |  |  |  |
| MSA  *Training Day 1*  *Training Day 2*  *Training Day 3* | 138 | .3 | .56 |  |  |  |  |  |
|  | 121 | .74 | .35 |  |  |  |  |  |
|  | 120 | .52 | .41 |  |  |  |  |  |
| Online Gain  *Training Day 1*  *Training Day 2*  *Training Day 3* | 121.5 | .47 | .42 |  |  |  |  |  |
|  | 121 | .72 | .37 |  |  |  |  |  |
|  | 121 | .72 | .36 |  |  |  |  |  |
| Offline Gain  *Consolidation 1*  *Consolidation 2* | 138.5 | .28 | .53 |  |  |  |  |  |
|  | 99 | .58 | .42 |  |  |  |  |  |

Note: *η_p_^2^*: partial eta-squared, *BF_10_:* Bayes Factor for evidence of the null hypothesis, significant values marked in bold.

STABLE 3: Statistical summary tDCS effects on arc pointing task learning younger adults.

| **Parameter** | **Factor 1: Condition main effect** | | | | **Interaction:**  **Condition x Day (x Trial)** | | | |
| --- | --- | --- | --- | --- | --- | --- | --- | --- |
|  | ***F*** | ***p*** | ***η_p_^2^*** | ***BF_10_*** | ***F*** | ***p*** | ***η_p_^2^*** | ***BF_10_*** |
| Movement Time  *Training Trials*  *Transfer Trials* | 4.05 | .05 | .13 | 1.55 | 1.35 | .08 | .05 | .004 |
|  | .65 | .43 | .02 | .53 | .32 | .64 | .01 | .22 |
| Accuracy  *Training Trials*  *Training Trials with CV (tr1)*  *Transfer Trials* | **15.65** | **<.001** | **.36** | **39.45** | 1.006 | .46 | .04 | .004 |
|  | **10.91** | **.003** | **.29** | **18.47** | .94 | .58 | .03 | .004 |
|  | .9 | .35 | .03 | .44 | .64 | .53 | .02 | .26 |
| Mean Spatial Variability | .47 | .5 | .02 | .37 | .13 | .75 | .01 | .38 |
|  |  |  |  |  |  |  |  |  |
|  |  |  |  |  |  |  |  |  |
|  | **Mean comparison:**  **(a-tDCS, s-tDCS)** | | |  |  |  |  |  |
|  | ***U*** | ***p*** | ***BF_10_*** |  |  |  |  |  |
| MSA  *Training Day 1*  *Training Day 2*  *Training Day 3* | 85 | .26 | .45 |  |  |  |  |  |
|  | 131.5 | .44 | .47 |  |  |  |  |  |
|  | 155 | .08 | 1.03 |  |  |  |  |  |
| Online Gain  *Training Day 1*  *Training Day 2*  *Training Day 3* | 152 | .1 | .82 |  |  |  |  |  |
|  | 123 | .66 | .35 |  |  |  |  |  |
|  | 116 | .86 | .35 |  |  |  |  |  |
| Offline Gain  *Consolidation 1*  *Consolidation 2* | 70 | .08 | 1.05 |  |  |  |  |  |
|  | 103 | .7 | .37 |  |  |  |  |  |

Note: *η_p_^2^*: partial eta-squared, *BF_10_:* Bayes Factor for evidence of the null hypothesis, significant values marked in bold.
